# Predictive Modeling of Yield Sooting Index Using Machine Learning with Uncertainty Estimation

**DOI:** 10.1021/acsomega.5c00042

**Published:** 2025-06-10

**Authors:** Zied Hosni, Xike Chen, Sofiene Achour, Fatma Saadi

**Affiliations:** a 4919 University College London, Gower Street, London WC1E 6BT, United Kingdom; b Research Unit of Modeling in Fundamental Sciences and Didactics, IPEIEM, University of Tunis El Manar, PO Box 254, El Manar 2, Tunis 2096, Tunisia; c Center for Research in Microelectronics and Nanotechnology (CRMN), Technopo le de ″Sousse Novation″, City Sahloul BP 334 SahloulSousse 4054,Tunisia; d Department of Chemistry, College of Science, 158223 Northern Border University, Arar 91431, Saudi Arabia

## Abstract

This study explores the development of two predictive models for the yield sooting index (YSI) of various fuels using the advanced capabilities of machine learning (ML), particularly multilayer perceptron (MLP) networks. Quantitative structure–property relationship (QSPR) methodology, which connects molecular structures with fuel properties, enables accurate predictions of fuel behavior, including YSI, kinematic viscosity, ignition temperature, and cetane and octane numbers. By utilizing feature selection techniques such as Gini importance and genetic algorithms, we identified key molecular descriptors that significantly impact YSI. Remarkably, the genetic algorithm model outperformed the Gini importance model by effectively reducing autocorrelation among features, thereby enhancing the accuracy of predictions. The reliability of these models was further validated through uncertainty estimation at different significance levels, which provided deeper insights into their performance. Additionally, we identified a strong relationship between certain 2D matrix-based descriptors and YSI, which offers a fresh perspective on predicting fuel properties. This comprehensive approach, incorporating rigorous data preprocessing, feature selection, and hyperparameter tuning, demonstrated the robustness of the developed predictive models. This work highlights the potent synergy between ML and QSPR theory in advancing the prediction of fuel properties. It not only refines current predictive models but also sets the stage for future computational advancements in fuel research, which contributes to the broader goal of developing sustainable and efficient fuel alternatives.

## Graphical abstract

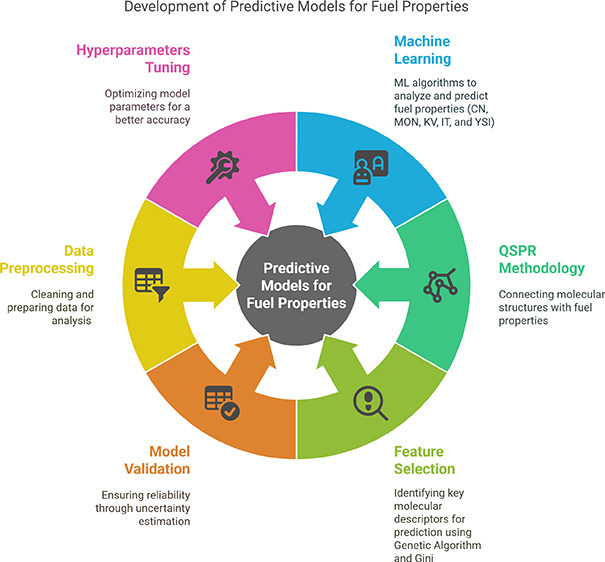

## 1 Introduction

The study of fuel-sooting tendency is crucial due to its direct impact on combustion efficiency, environmental pollution, and human health. Soot emissions from combustion processes contribute significantly to global warming and air pollution, leading to adverse health effects.^1–3^ Understanding and mitigating soot formation is essential for the development of cleaner and more efficient combustion technologies. Research on soot dynamics has aided in identifying biofuel candidates that reduce soot production,^4,5^ to optimize engine performance, and to meet stringent emission standards. By investigating the sooting propensity of different fuels, such as biofuels and solid fuels, researchers can assess their environmental impact and potential for reducing harmful emissions.

Traditional methods like the Method of Moments (MOM) have been utilized to study sooting tendencies in flames, but they have struggled to characterize continuous particle size distributions, hindering accurate particle oxidation assessment.^6^ To address this, the Split-based Extended Quadrature Method of Moments (S-EQMOM) has emerged as a robust alternative, which allowed the reconstruction of continuous particle number density functions through kernel density functions (KDF) superimposition.^7^ Furthermore, a quantitative structure–property relationship model has been developed to predict the threshold sooting index (TSI) of various hydrocarbon mixtures, by integrating linear blending rules and regression techniques.^8^ However, these traditional methods often come with computational challenges and limitations in accurately modeling soot formation. This prompted the development of more efficient and accurate approaches like ML and neural networks to estimate soot characteristics in flames.^9,10^ Indeed, ML enhanced the prediction of fuel-sooting tendencies compared to traditional methods by offering more accurate and reliable models. For instance, the use of random forest (RF) ensemble models based on large data sets has shown high prediction accuracy for fuel consumption and emission rates in urban areas.^11^ In addition, a data-driven model augmentation framework, such as weakly coupled Integrated Inference and Machine Learning (IIML), significantly improved the predictive accuracy of physical models by inferring corrections to the model structure and transforming them into corrective forms.^12^

A novel approach called U-net has been employed to retrieve local soot temperature and volume fraction fields from soot radiation measurements. It showed high prediction accuracy and robustness to noise.^13^ Additionally, a Bayesian optimized back-propagation neural network (BPNN) has been developed for diagnosing key parameters in sooting flames, achieving accurate predictions of soot temperature, volume fraction, and primary particle diameter.^14^ These ML techniques offer efficient and cost-effective ways to enhance the understanding of combustion processes, optimize performance, and reduce emissions, which ultimately contributed to the sustainability of combustion systems.^15^

Recent advances in machine learning (ML) have opened new avenues for predicting fuel sooting tendencies with high precision.^16^ Several studies have demonstrated the efficacy of quantitative structure–property relationship (QSPR) models and artificial neural networks (ANNs) in estimating sooting indices. For example, St. John et al.^17^ developed a robust QSPR model that correlates molecular structure with sooting tendency, achieving high predictive accuracy across diverse hydrocarbons. Li et al.^18^ further compared conventional ML methods with deep learning (DL) approaches, highlighting that automated feature extraction via convolutional neural networks can capture complex nonlinear relationships inherent to fuel chemistry. In parallel, models developed by Qasem et al. and Abdul Jameel successfully applied ANN techniques to predict threshold and yield sooting indices for oxygenated fuels, demonstrating the significant role that functional group descriptors play in sooting behavior.^19,20^ More recently, Chen et al.^21^ presented a ML framework tailored for spark-ignition engine applications, while Pfefferle et al. provided an extensive review on sooting tendencies for sustainable fuel design. Despite these important contributions, our work distinguishes itself by not only further refining prediction accuracy through a multilayer perceptron network enhanced with dual feature selection strategies (employing both Gini importance and genetic algorithms) but also by incorporating a rigorous uncertainty estimation framework. This dual approach enhances model robustness and interpretability, providing quantitative confidence in predictions applied to novel fuel compositions; thereby moving the research forward toward more reliable and practical tools for sustainable fuel development.

The design of ML models to predict the sooting tendency of fuels requires key considerations, namely the need for accurate emission modeling due to stringent standards.^14^ This necessitates the use of experimental data to parametrize virtual engines and develop accurate physical models.^7^ Feature selection methods like LASSO could aid the development of computationally efficient soot models with good precision.^22^ Model validation is crucial, with the use of K-means clustering to categorize feature sets and methods based on performance for selecting the best approach.^23^ Additionally, the flexibility and robustness of models were quantitatively evaluated by introducing noise during training and testing.^24^

Despite advancements in modeling soot formation, there remains a need for more accurate and computationally efficient models to predict the sooting tendencies of various fuels. Therefore, the primary objective of this study is to develop and compare two machine learning models (a Gini importance-based model and a genetic algorithm-based model) for predicting the YSI of fuels. Additionally, we incorporate uncertainty estimation to assess the reliability of our predictions.

## 2 Computational Method

### 2.1 Data Set Extraction

A set of 558 molecules of experimentally determined YSI data was obtained from YSI Database, which was constructed by Pfefferle Research Group.^25^ Their CAS numbers were transformed into Simplified molecular-input line-entry system (SMILES), which is a method to encode chemical formulas as simple text strings.^26^ The Chemical Identifier Resolver implemented in KNIME (Alvascience Ltd.) was used to convert CAS numbers to SMILES.^27^ We then performed data cleaning by removing duplicates and compounds with missing values to ensure data set integrity. The molecular descriptors were then computed using alvaDesc, which included a comprehensive set of 5666 descriptors that covered various aspects of both 2D and 3D information, which included as constitutional, topological, and pharmacophore features.^28^ A full list of the calculated molecular descriptors is available on the Alvascience Web site (http://www.alvascience.com).

### 2.2 Predictive Model Deployment

The architecture of the present model is based on Enhanced Chemical Network (ECNet), which is an open-source Python package designed for cost-effectively predicting essential fuel properties of potential next-generation biofuels.^29^ By employing neural networks to enhance precision, ECNet could forecast various fuel properties like Cetane Number (CN), YSI, Research Octane Number (RON), and Motor Octane Number (MON) through the use of Quantitative Structure–Property Relationship (QSPR) input parameters. Figure 1 presents a pipeline that describes the prediction workflow. Initially, experimentally determined fuel properties were obtained from the YSI Database (constructed by the Pfefferle Research Group^25^), and their molecular descriptors were generated using the alvaDesk tool from Alvascience. Then, two feature selection methods were applied to filter unnecessary features, accelerate computing, and increase accuracy. The preprocessed data set was partitioned into three subsets: 65% for training, 10% for testing, and 25% for calibration to facilitate uncertainty estimation. This specific split was determined through preliminary experiments that balanced the need for ample training data against the requirement for independent evaluation and calibration. In order to minimize the impact of random fluctuations, particularly for properties with smaller sample sizes such as MON (306 samples), we performed cross-validation. In each iteration, the data was randomly divided according to the 65/10/25 scheme, and the final performance metrics are presented as averages over these multiple iterations. This robust evaluation strategy confirmed that our model’s performance estimates are reliable despite the challenges posed by smaller data sets. The regression model was built based on a multilayer neuronal network. Finally, uncertainty estimation was performed through conformal prediction. The model was implemented within the graphical programming framework Knime.^30^

**Figure 1:**
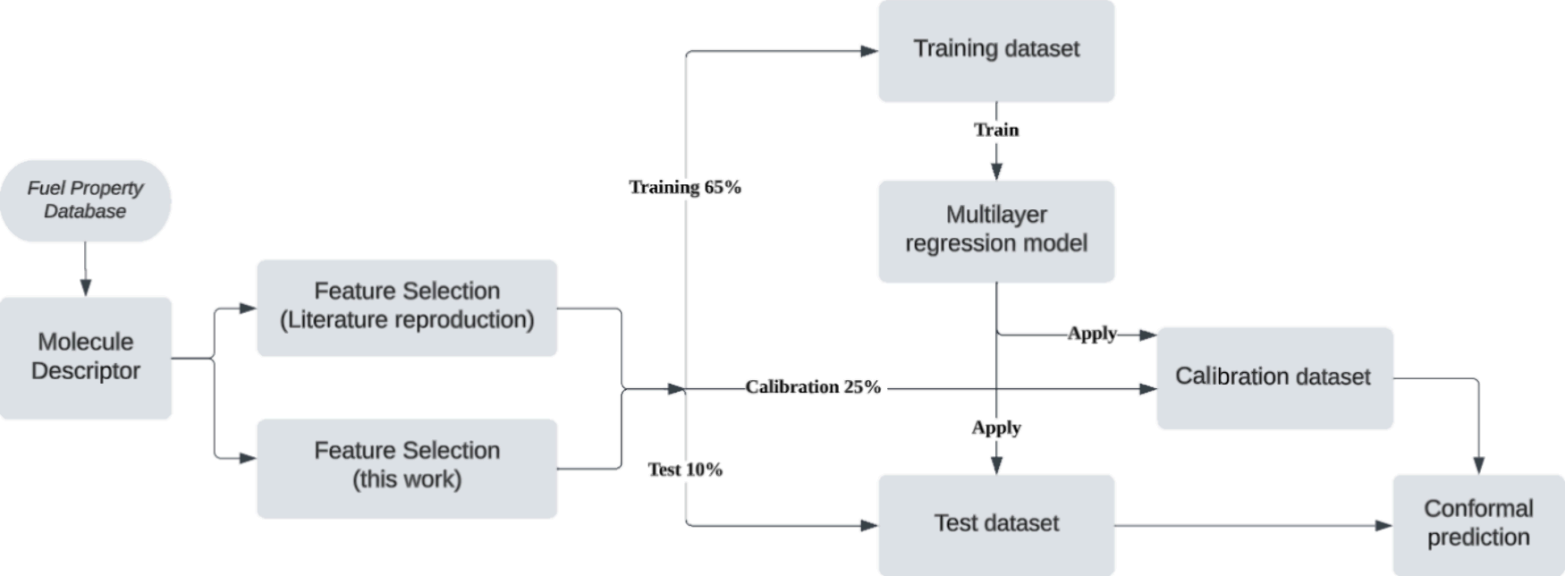
Flow diagram of the prediction pipeline used to predict YSI.

### 2.3 Feature Selection

Two dimensionality reduction methods were used to convert the molecular descriptors and reduce the number of features employed to train the predictive model. The first used Gini importance as a criterion to select features. This allowed the reproduction of feature selection performed in ECNet^29^ using KNIME platform.^31^ The second was a combination of multiple feature reduction techniques that include a variance filter, correlation filter, and a genetic algorithm-based feature selection loop. These two methods were called Gini^32^ for Gini feature importance model and GA^33,34^ for the genetic algorithm model, respectively. Gini importance was chosen due to its effectiveness in measuring the significance of features in tree-based models by evaluating the decrease in impurity. Genetic algorithms were employed for their ability to optimize feature subsets by simulating natural selection processes, which is particularly advantageous for handling high-dimensional data sets. To further enhance the understanding of feature importance, the subsequent step involved the application of the Random Forest feature selection process. This approach permitted a systematic examination of individual features based on their impact on improving or hindering the performance of a Random Forest algorithm. The feature selection strategy based on genetic algorithm employed a population size of 5 and maximum number of generations equal to 20 for computing efficiency.

### 2.4 Multilayer Perceptron Regression Model

MLP was implemented using KNIME Keras integration.^35,36^ The MLP architecture was optimized using the Adam optimization function to allow precise adjustments of learning rate and decay. This optimization facilitated a tailored approach that enabled the fine-tuning of parameters. The model’s performance was gauged using mean squared error (MSE) as the loss function described in EQ 1, recognized for its effectiveness in regression analyses.

$$MSE=\frac{1}{N}\overset{N}{\underset{i=1}{\sum }}{\left(y_{i}-{ŷ}_{i}\right)}^{2}$$ 1

where *N*, *y*_*i*_ *ŷ*_*i*_ is the number of compounds, the measured value, the model predictive value, respectively.

For hyperparameters optimization, we applied Bayesian Optimization with Tree-structured Parzen Estimation (TPE), as described in Table 1, which lists the hyperparameters and their ranges. This process involved a two-phase strategy: an initial ‘warm-up’ phase for random parameter evaluation, followed by a systematic search for optimal hyperparameters, guided by the coefficient of determination (R^2^) as the performance metric, illustrated in EQ 2.

$$\begin{array}{c} R^{2}=1-\frac{MSE\left(model\right)}{MSE\left(baseline\right)}=1-\frac{\frac{1}{N}\overset{N}{\underset{i=1}{\sum }}{\left(y_{i}-{ŷ}_{i}\right)}^{2}}{\frac{1}{N}\overset{N}{\underset{i=1}{\sum }}{\left(y_{i}-\overset{®}{y_{i}\;}\right)}^{2}} \end{array}$$ 2

where $\overset{®}{y_{i}\;}$ is the mean of measured values described in EQ 3.

$$\overset{®}{y_{i}\;}=\frac{1}{N}\overset{N}{\underset{i=1}{\sum }}y_{i}$$ 3

**Table 1:** Hyperparameters and Range Used to Optimize the MLP Model

hyperparameter	range
number of hidden layers	1–5
number of neurons per layer	1–1024
learning rate	0.1–0.5
learning decay	0–0.1

The exploration of parameter combinations was performed to determine the most suitable hyperparameters within the predefined range. This ensured a systematic and rigorous approach to model optimization. The default configuration for the search strategy included 100 iterations and 20 warm-up rounds. This highlighted the nuanced balance between optimization efficiency and the adaptability of the neural network. We utilized the Adam optimizer while the batch size was set to 32, and the model was trained for 100 epochs. To ensure the robustness of the selected hyperparameters, we used a 5-fold cross-validation scheme, with the entire optimization process repeated across multiple random splits. The final hyperparameter values, comprising the number of hidden layers, neurons per layer, learning rate, and learning decay, were chosen as the average of the best-performing configurations across the folds. These consistent results across the folds indicate that the optimized hyperparameters are stable and not overly sensitive to the particular data partitioning. The best hyperparameter values after the optimization are 4 hidden layers, 237 neurons, learning rate of 0.241 and learning decay of 0.013.

### 2.5 Uncertainty Estimation

Our study implemented uncertainty estimation using the KNIME conformal regression toolbox developed by Redfield AB, based on the foundational work of Norinder et al.,^30,37^ which employs conformal prediction methods to provide prediction intervals with a specified confidence level. Conformal predictors generated a prediction region Γ­(ϵ,i) for each test instance x_i_, which ensured that the true target y_i_ is contained within this region with a probability of 1- ϵ, and thereby defined the error rate of the model. These predictors maintained automatic validity, which meant that, over an extensive data set, the observed error rate aligned with ϵ, by assuming that data are independently and identically distributed. The efficiency of conformal predictors, demonstrated by the compactness of prediction regions, is crucial, especially for regression tasks where narrower intervals indicate more precise predictions. Our workflow focused on optimizing the error rate and the sensitivity parameter β. Absolute error (AE), expressed as |*y*_*i*_–*ŷ*_*i*_|, served as the measure of prediction difficulty σ, informing the interval size and error rate metrics critical for conformal regression. This trade-off between interval size and error rate s pivotal for precise predictions, which necessitated a balance that ensured both accuracy and confidence in the predicted intervals. In optimizing the process, we scrutinized a range of β values from 0.25 to 1, with a step size of 0.25, to find the optimal balance that influences prediction intervals’ size and error rate. Simultaneously, we adjusted the error rate within a 0 to 0.25 range, incrementally increasing by 0.05, to modulate the prediction intervals accordingly. This systematic optimization, depicted in Figure 2, outlines the construction of conformal predictors for regression, to select the most suitable parameters for our predictive model. There are also two main metrics for conformal regression: error rate, which is the experimental one that comes from predictions; the interval size, that describes the band within which the predictionsare located. The interval size is expected to decrease with an increase of the error rate. Hence, a trade-off should be made between these two metrics.

**Figure 2:**
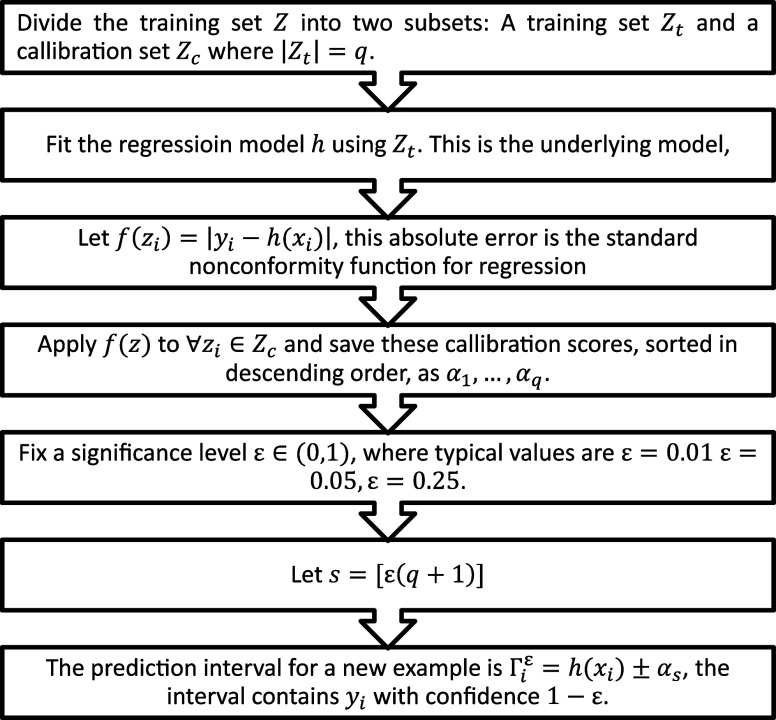
Procedure for constructing conformal predictor for regression.

## 3 Results and Discussion

### 3.1 Dimensionality Reduction

To prepare the model for the training step, 220 features were identified via the Random Forest Feature Selection Loop and subsequently normalized employing Z-score normalization. Figure 3 presents a correlation heatmap comparing two distinct feature selection methodologies. Specifically, Figure 3a, derived from the literature reproduction method, and Figure 3b, developed using our novel hybrid approach, both elucidated the impact of different preprocessing strategies on the correlation structure among features. The comparative analysis of these heatmaps highlighted a significant difference in the correlation patterns across the two methods. Figure 3a exhibits a higher density of red zones, signaling a strong presence of autocorrelated features. This suggested that the literature-based method may retain more interrelated features. On the other hand, Figure 3b, which represents the hybrid selection approach, showed a more varied color distribution, which indicated a reduced level of feature autocorrelation. This variance in the color spectrum and intensity between the two heatmaps underscored the effectiveness of the hybrid method in diminishing autocorrelation among features, thereby potentially enhancing the robustness and predictive performance of the resulting model. The analysis demonstrated how the choice of feature selection method can significantly alter the underlying feature correlation landscape, and impact the model’s overall interpretability and efficacy.

**Figure 3:**
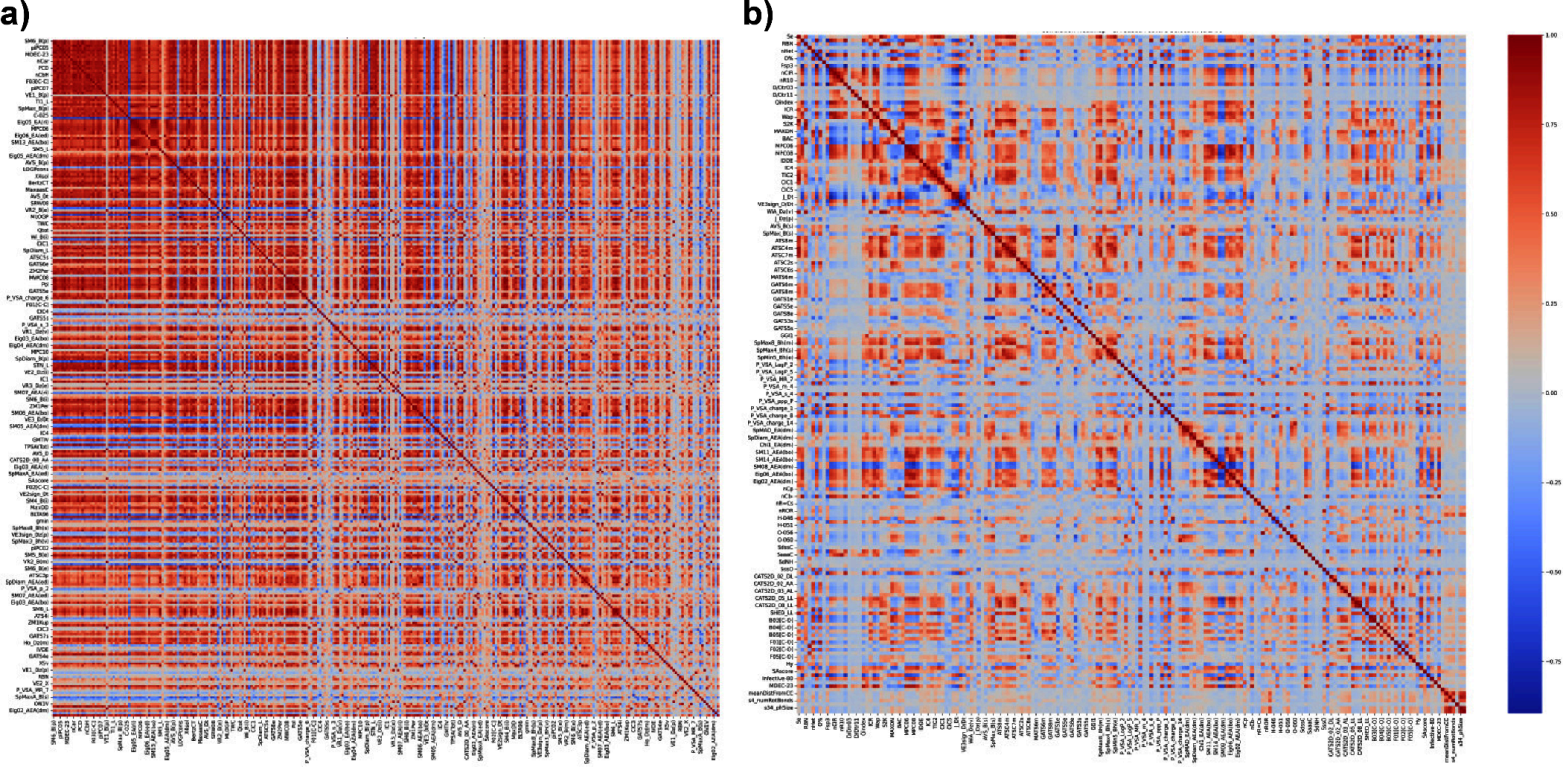
: Correlation heatmap after preprocessing. (a) Literature reproduction. (b) Hybrid method.

### 3.2 Model Performance

In this study, the robustness of the predictive models was quantitatively assessed using both the coefficient of determination (R^2^) and an analysis of the prediction error distribution, with particular attention to outliers. Although all models achieved high R^2^ values, indicating that most predictions fell within the experimental uncertainty, the Baseline and GA models each produced an extreme outlier (predicting near-zero for a compound with a measured YSI near 200), an issue not observed with the Gini model. This outlier analysis stresses the enhanced robustness of the Gini model in reliably capturing fuel sooting tendencies. Moreover, confidence intervals were used to assess the robustness of our prediction for both the Gini and GA models. To further elucidate the performance dynamics of the predictive systems, an examination was conducted across three distinct models, each underpinned by a MLP architecture. The choice of MLP was based on its proficiency to delineate complex data relationships, and to offer a robust basis for predictive analysis. Although these models are unified in their core computational configurations, their performance nuances were attributed to varied preprocessing methodologies. The baseline model operated on a comprehensive data set encompassing 558 molecular entities, where each characterized by 5666 descriptors, foregoing any specialized feature selection mechanism. Contrarily, the Gini feature selection-based model narrowed its analytical focus, leveraging Gini importance metrics to distill the feature set down to 237 descriptors. This approach not only reduced dimensionality but also enhanced model efficiency and interpretability.^38^ The third model, employing a genetic algorithm for feature selection, represented a more sophisticated strategy. This model merged genetic algorithm principles with conventional feature selection, by iteratively refining and optimizing the descriptor pool. This selection mechanism was designed to maximize the predictive accuracy and generalizability of the model by systematically eliminating redundancy and preserving the most informative features.

The comparison of the three models revealed that the genetic algorithm (GA) based model exhibited an enhanced performance, particularly in terms of *R* ^2^_test_, when juxtaposed with the baseline model. Although the baseline and GA models displayed similar accuracies on the training set, the Gini model outperformed others on the test set, by achieving an *R* ^2^_test_ of 0.979 with a standard deviation of 0.005, as evidenced in Figure 4. These findings suggested that the four-layer MLP model was adept at handling data sets of varying complexities. Further application of the models across different properties, as detailed in Table 2, consistently showed that the GA model surpassed the Gini model in terms of *R* ^2^ metric. Notably, the GA method yielded a feature set that was 30% to 45% smaller than that obtained via the Gini method for predicting fuel properties such as CN (cetane number), MON (motor octane number), KV (kinematic viscosity), and IT (ignition temperature), which indicated a more efficient feature selection process. The heatmap in Figure 3 corroborates that the GA model’s elimination of autocorrelated features has augmented prediction accuracy.

**Figure 4:**
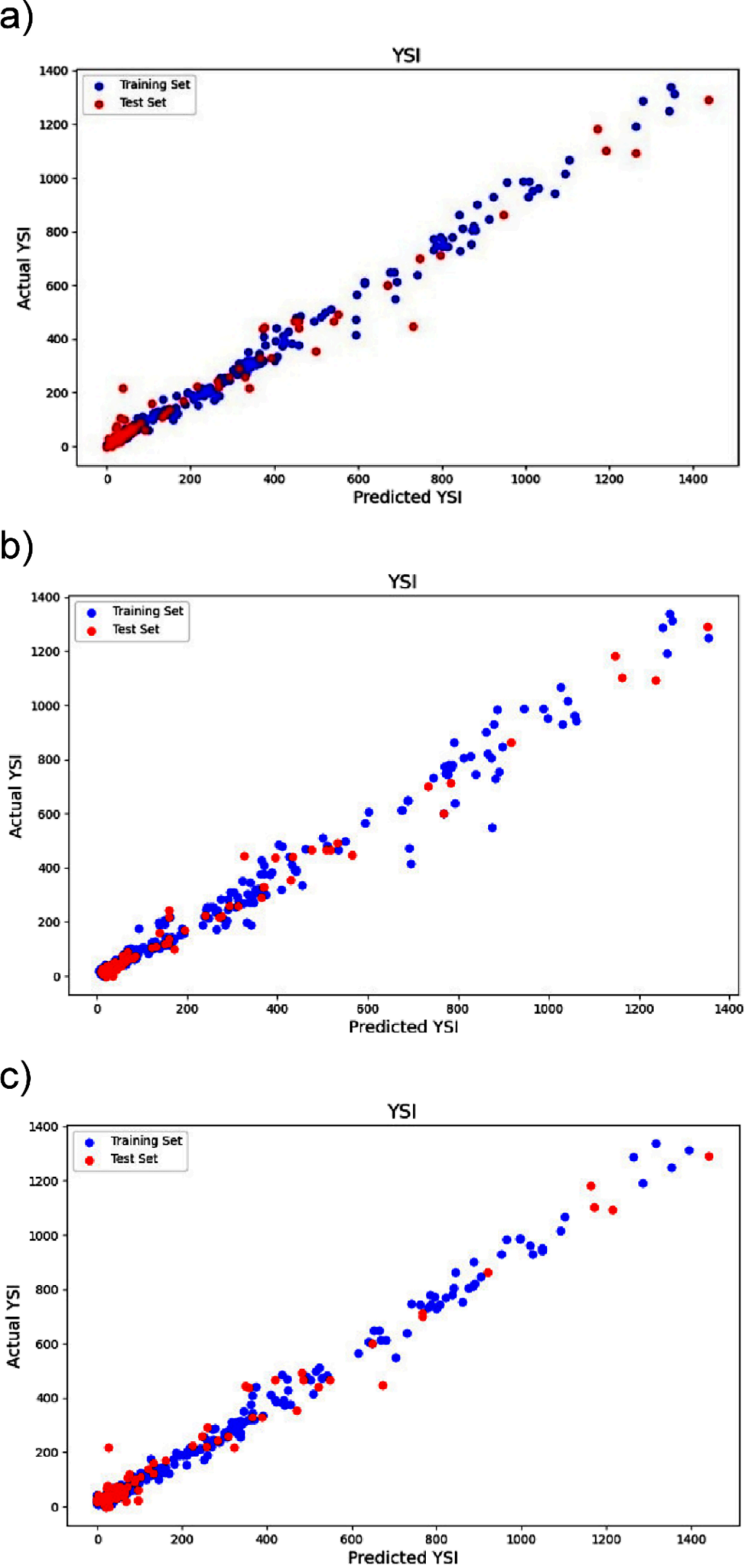
(a) YSI prediction result for baseline model, *R*_train_^2^ = 0.988, *R*_test_^2^ = 0.962, (b) YSI prediction result for Gini model, *R*_train_^2^ = 0.976, *R*_test_^2^ = 0.979, (c) YSI prediction result for GA model, *R*_train_^2^ = 0.988, *R*_test_^2^ = 0.967.

**Table 2:** Comparison of GA and Gini Model through CN, MON, KV, IT, and YSI^a^

		GA		Gini	
property name	compound No.	feature no.	*R* ^2^	features no.	*R* ^2^
CN	460	177	0.84	260	0.81
MON	306	154	0.801	280	0.78
KV	213	154	0.86	281	0.86
IT	377	157	0.70	270	0.62
YSI	558	220	0.97	237	0.97

a *R* ^2^ is based on the test set.

Sample size also emerged as a significant factor to influence prediction accuracy. For instance, with the largest data set size of 558 samples, both GA and Gini models predicted YSI with an *R* ^2^ of 0.97. However, for KV with only 213 samples, the *R* ^2^ dropped to 0.86 for both models. This trend suggested that while sample size and feature optimization were crucial, the intrinsic complexity of the target property also played a pivotal role in model performance. Despite similar feature processing for MON and KV, and a higher sample count for MON, its *R* ^2^ was unexpectedly lower than that of KV, which hinted at underlying complexities that might be affecting the predictive accuracy. This anomaly presented an intriguing avenue for future work to unravel the factors influencing prediction performance across different properties. It was evident that the genetic algorithm (GA) model consistently achieved higher R^2^ values across various properties even with a notably reduced feature set. This efficiency in feature use was particularly pronounced in comparison with the Gini model, where the GA model used 30% to 45% fewer features for properties like CN, MON, KV, and IT. This reduction in features suggested that the GA method was particularly effective in identifying and retaining only the most predictive features. In terms of sample size, the YSI property, with the largest data set of 558 samples, showed a high prediction accuracy (R^2^=0.97) for both GA and Gini models, which confirmed the potential of these models to handle large data sets effectively. Conversely, the KV property, with only 213 samples, exhibited a lower R^2^ of 0.86, which highlighted the challenges associated with smaller data sets. Interestingly, despite the significant difference in the number of samples between MON (306 samples) and KV (213 samples), the R^2^ values did not follow the expected trend of higher R^2^ with more samples. Instead, MON’s coefficient of determination was lower than that of KV, which is consistent with expectations since kinematic viscosity is a physical property more directly linked to molecular structure, whereas motor octane number is a kinetic property that depends on the reaction mechanisms and is inherently more challenging to predict. Therefore, the detailed examination of the performance metrics across different properties elucidated the nuanced interplay between feature selection efficiency, sample size, and the intrinsic characteristics of the target variables. Furthermore, these insights stressed the necessity for a tailored approach in predictive modeling, where both the selection of features and understanding the data set’s underlying structure were critical for optimizing model performance. The baseline model (Figure 4a) exhibited a high training R^2^ value, which indicated strong learning from the training data set, yet the lower testing accuracy suggested a potential overfitting. Conversely, the Gini model (Figure 4b) showed a balanced accuracy between the training and testing phases. Thus, this indicated a well-generalized model. Tin addition, the GA model (Figure 4c) followed the baseline in terms of a high training accuracy but with a slight reduction in testing accuracy. This thus pointed toward the need for model refinement to enhance its generalization.

In terms of the learning dynamics of the MLP, Figure 5 offers an insightful overview of its training trajectory. A sharp decline in loss at the initial stages of training indicates the model’s rapid adaptation and understanding of the data’s fundamental patterns. As training proceeded, the curve flattened, which suggested a transition from broad learning to more detailed and nuanced error reduction. This phase of training was characterized by the model’s incremental improvements, and a refinement of its predictive accuracy through gradual learning and optimization of its parameters.

**Figure 5:**
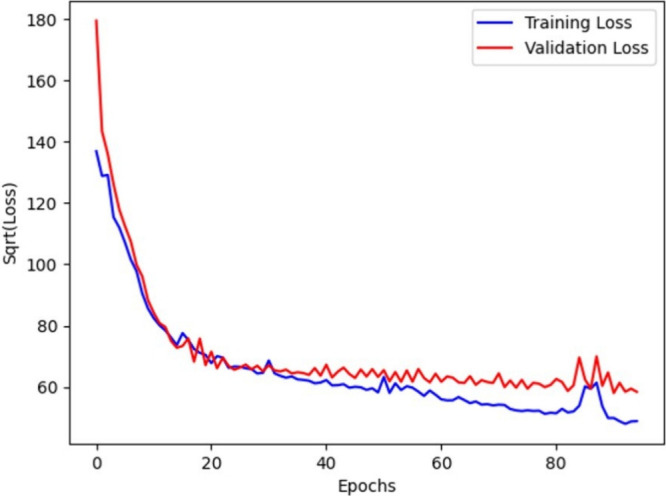
Learning curve of MLP regression model to predict YSI.

### 3.3 Model Uncertainty Estimation

Accurately estimating model uncertainty is essential for understanding its predictive reliability, especially in the context of Yield Strength Index (YSI) predictions. In this section, we examine the prediction intervals generated using two models—GA and Gini—at different confidence levels (99%, 95%, and 75%). These prediction intervals highlight the model’s performance under varying levels of confidence, with wider intervals indicating greater uncertainty in the predictions, and narrower intervals reflecting higher confidence. Figure 6 provides a comprehensive visualization of prediction accuracy across confidence levels (99%, 95%, and 75%), showing the intervals between predicted and actual YSI values. As expected, higher confidence levels lead to wider intervals, reflecting the model’s caution in capturing the true values with more certainty. Conversely, lower confidence levels lead to narrower intervals, as the model becomes more tolerant of error in exchange for a tighter prediction range.

**Figure 6:**
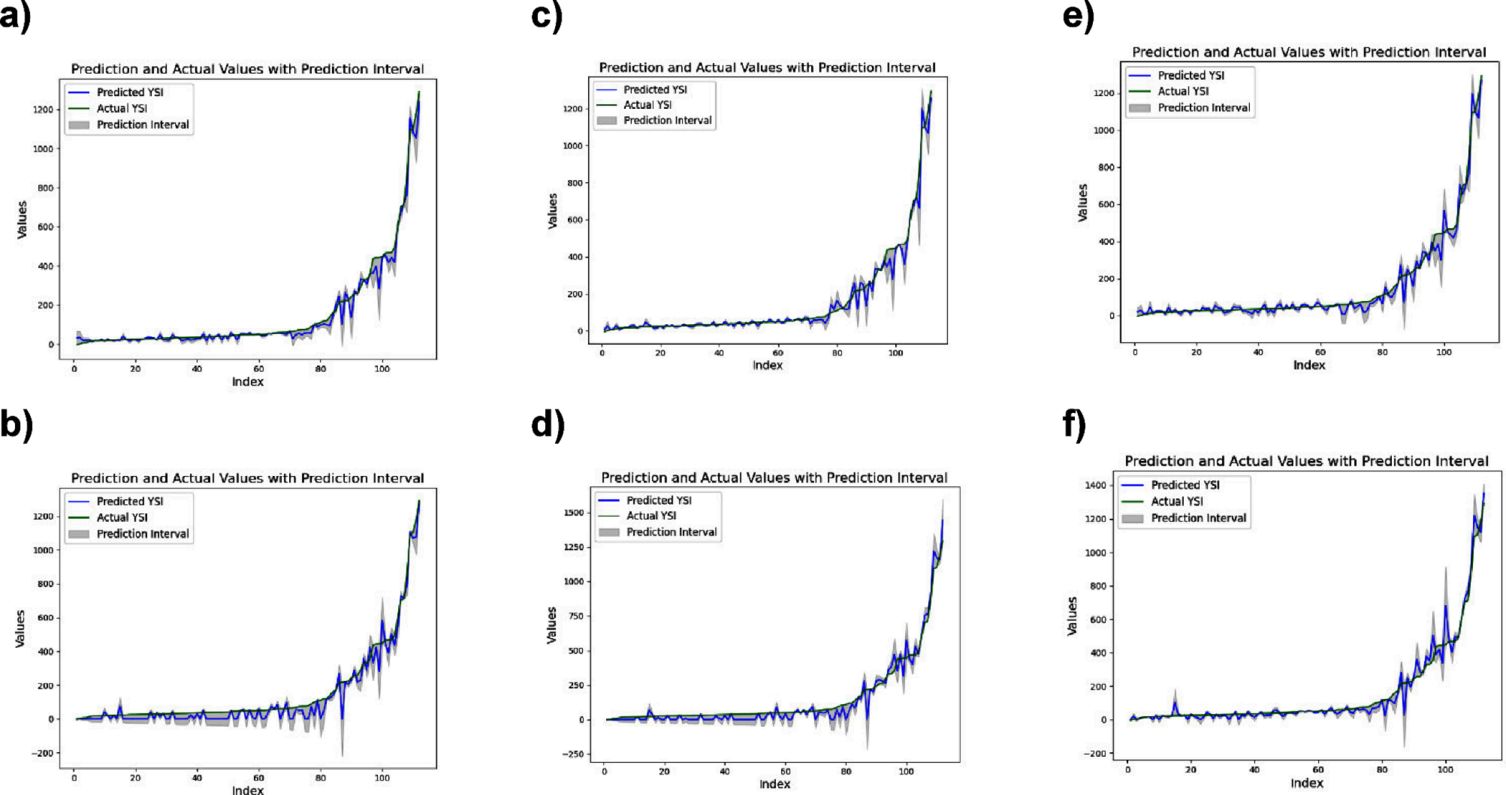
Interval between prediction and actual YSI at (a) 99% confidence level and Gini model, (b) 99% confidence level and GA model, (c) 95% confidence level and Gini model, (d) 95% confidence level and GA model, (e) 75% confidence level and Gini model, and (f) 75% confidence level and GA model.

At the 99% confidence level (Figures 6a and 6b), the intervals between predicted and actual YSI values are wider. This reflects a high degree of confidence in the model’s predictions, meaning that the model is expected to capture the true YSI values with minimal error. The wider intervals at this level suggest that the model is more cautious, accounting for more uncertainty to ensure the true value is likely within the interval. As we move to the 95% confidence level (Figures 6c and 6d), the prediction intervals narrow slightly, which is in line with expectations given the reduced confidence threshold. Although the intervals are somewhat tighter than at 99%, they still encompass most of the actual YSI values, indicating that the model remains reliable with a strong balance between precision and confidence. At the 75% confidence level (Figures 6e and 6f), the prediction intervals narrow significantly, reflecting reduced uncertainty in the model’s predictions. The tighter intervals reflect a lower level of confidence, where the model is more certain of its predictions but accepts a higher risk of missing the true YSI values. Despite this reduced coverage, the majority of actual YSI values still fall within these narrower intervals, suggesting that the model performs adequately, though with less caution in its predictions.

When comparing the GA and Gini models, some differences in interval sizes emerge. At both the 99% and 95% confidence levels, the Gini model shows slightly narrower intervals than the GA model, indicating higher precision and reliability at these levels. However, as the confidence level drops to 75%, the intervals narrow for both models, but the Gini model continues to maintain a slight advantage in terms of interval compactness. This suggests that the Gini model handles prediction variability more effectively than the GA model under less stringent conditions, providing tighter intervals without sacrificing reliability.

The calibration plots displayed in Figures 7a and 7b elucidate the relationship between the predicted and actual error rates for the models under consideration. In an ideal scenario, as represented by the theoretical error rate (i.e., orange line in the graph), the calibration plot would exhibit a linear trend with a slope of 1. This means a perfect alignment between predicted probabilities and actual outcomes. The expected error rate, often derived from prior knowledge or assumptions, served as a benchmark for assessing the model’s anticipated performance on novel data. In the case of the Gini model, the actual error rates were consistently lower than the theoretical ones for significance levels ranging from 0 to 0.7. This observation suggested that the Gini model’s predictive accuracy exceeded the initial expectations within this range, which indicated a robust performance. Conversely, for the GA model, the actual error rates surpassed the theoretical ones at lower significance levels, and implied a potential overestimation of the model’s performance in these instances. However, as the significance level increased, this trend did not persist and suggested that the GA model’s performance aligned more closely with expectations. Therefore, this ensured its reliability. In addition, these calibration plots revealed that both the GA and Gini models could make accurate predictions, but with some nuances in performance across different significance levels. Interestingly, the discrepancies between the actual and theoretical error rates underscored the importance of continuous model evaluation and adjustment to refine predictive accuracy.

**Figure 7:**
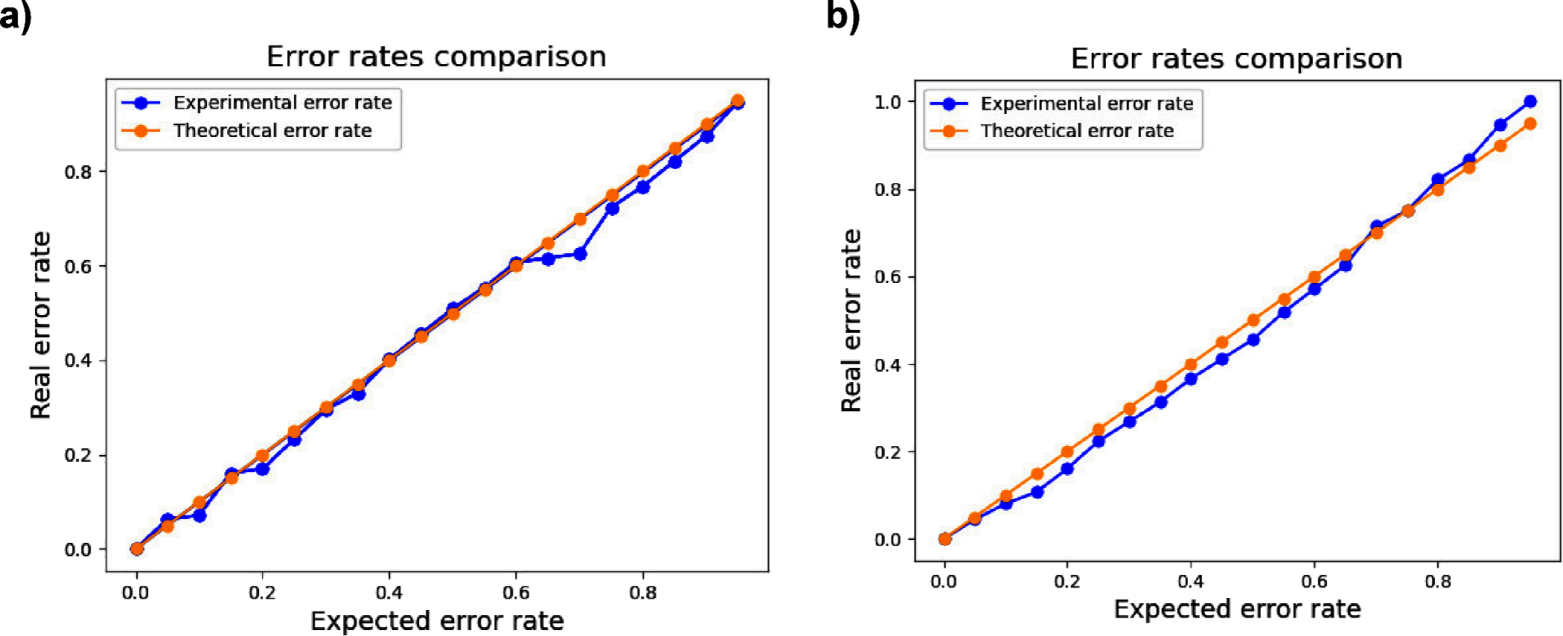
Calibration plot corresponding to (a) GA model and (b) Gini model.

### 3.4 Molecule Descriptor Analysis

The Gini importance method provided a quantitative assessment of descriptor significance, enabling a detailed comparison of each descriptor’s influence on the predictive model. Table 3 presents the top ten descriptors with the highest Gini importance scores, and notably, two prominent features, piPC04 and piPC05, are categorized as molecular multiple path counts. These descriptors exhibit nearly identical importance scores, around 0.013, and play a crucial role in defining molecular connectivity by representing paths comprising N+1 atoms within a molecule. Such path-based descriptors are essential in capturing structural information about molecules that influences their physical and chemical properties, including Yield Strength Index (YSI). Recently, Kessler et al.^39^ introduced a multivariate exponential equation to correlate YSI with piPC descriptors, optimized through the Levenberg–Marquardt method for least-squares regression (LSR). This equation (shown below) models how molecular structure impacts YSI, with the summation running over various N-path orders and corresponding exponential coefficients:

$$\begin{array}{c} {\mathrm{YSI}}_{\mathrm{calc}}=\overset{5}{\underset{N=3}{\sum }}{\left(piPC\left(N\right)\right)}^{cN} \end{array}$$ 4

Here, the term (*piPC*(*N*))*^cN^* represents the exponential contribution of path count descriptors at different orders, N. The use of exponential fits is crucial because it allows the model to capture the rapid, nonlinear changes in molecular properties that occur as molecular complexity increases. This reflects broader findings where exponential relationships were preferred over linear or polynomial fits due to their ability to better capture molecular interactions in complex systems.^40,41^ While the piPC descriptors have been widely studied, an interesting and often overlooked set of descriptors is the 2D matrix-based descriptors (SM4_B­(p), SM5_B­(p), SM6_B­(p), SM6_B­(v), and SM5_B­(v)), which have emerged as significant contributors in our analysis. The N in SM­(N) refers to the spectral moment of order N, while B­(p) and B­(v) represent Burden matrices weighted by either mass or van der Waals volume, respectively. These descriptors are particularly insightful in quantifying molecular properties that are influenced by structural topology and atomic interactions. The high importance of descriptors such as SpMax1_Bh­(v) and piID suggests that electronic properties and molecular connectivity play significant roles in determining YSI. Understanding these relationships can guide the design of new fuel compounds with lower sooting tendencies.

**Table 3:** 10 QSPR Descriptors with the Highest Correlation to YSI

descriptor	importance	definition	group
SpMax1_Bh(v)	0.018	largest eigenvalue *n*; One of Burden matrix weighted by van der Waals volume	Burden eigenvalues
piID	0.015	conventional bond order ID number	walk and path counts
SM6_B(p)	0.014	spectral moment of order 6 from Burden matrix weighted by polarizability	2D matrix-based descriptors
piPC04	0.013	molecular multiple path count of order 4	walk and path counts
piPC05	0.013	molecular multiple path count of order 5	walk and path counts
SM4_B(p)	0.013	spectral moment of order 4 from Burden matrix weighted by mass	2D matrix-based descriptors
SM5_B(p)	0.012	spectral moment of order 5 from Burden matrix weighted by mass	2D matrix-based descriptors
SM6_B(v)	0.011	spectral moment of order 6 from Burden matrix weighted by van der Waals volume	2D matrix-based descriptors
CATS2D_02_LL	0.011	CATS2D lipophilic–lipophilic at lag 02	pharmacophore descriptors
SM5_B(v)	0.011	spectral moment of order 5 from Burden matrix weighted by van der Waals volume	2D matrix-based descriptors

The analysis of the top descriptors for YSI prediction highlights the complementary roles of path-count-based and 2D matrix-based descriptors in capturing critical aspects of molecular structure that influence soot formation. Path-count descriptors (e.g., piPC04 and piPC05) quantify the number of distinct atomic paths of defined lengths within a molecule, serving as proxies for the degree of molecular conjugation and aromaticity. Since aromatic structures are known to be precursors to the formation of polyaromatic hydrocarbons (PAHs), key intermediates in soot nucleation, a higher count of these paths often correlates with an increased sooting tendency.

In contrast, 2D matrix-based descriptors, such as those derived from Burden matrices (e.g., SM4_B­(p) and SM5_B­(p)), provide a holistic representation of molecular properties including mass distribution, polarizability, and van der Waals volume. These properties are intrinsically linked to the overall reactivity and stability of a molecule under combustion conditions. For instance, a descriptor that captures the distribution of atomic masses can reflect the molecule’s propensity to undergo pyrolytic fragmentation, which in turn influences the formation of soot precursors.

The integration of these descriptors into our machine learning framework not only improves prediction accuracy but also enhances the interpretability of the model. By quantitatively linking specific molecular features to sooting propensity, our approach provides a valuable tool for the rapid screening of fuel candidates. In real-world applications, this means that fuel formulations can be optimized to minimize soot production while maintaining desirable performance characteristics. This supports the development of next-generation sustainable fuels with reduced environmental impact.

While piPC descriptors focus on molecular connectivity through path counts, 2D matrix-based descriptors capture the influence of more global structural characteristics like mass, polarizability, and van der Waals volume. In this sense, the two sets of descriptors complement each other: piPC focuses on local, path-based structural details, whereas 2D descriptors offer a broader, matrix-based view of molecular topology. By incorporating both, the model leverages a synergistic relationship between detailed molecular paths and overarching structural characteristics, which enhances its overall predictive power. In our analysis, these 2D matrix-based descriptors exhibited strong correlations with YSI, as demonstrated in Figures 8a-8e. The exponential fits for each of these descriptors indicate their significant predictive relevance. The rationale behind using exponential fits, rather than linear or polynomial relationships, stems from the nonlinear nature of molecular behavior. Exponential models are particularly adept at capturing the rapid changes in molecular properties, such as YSI, as descriptor values increase.

**Figure 8:**
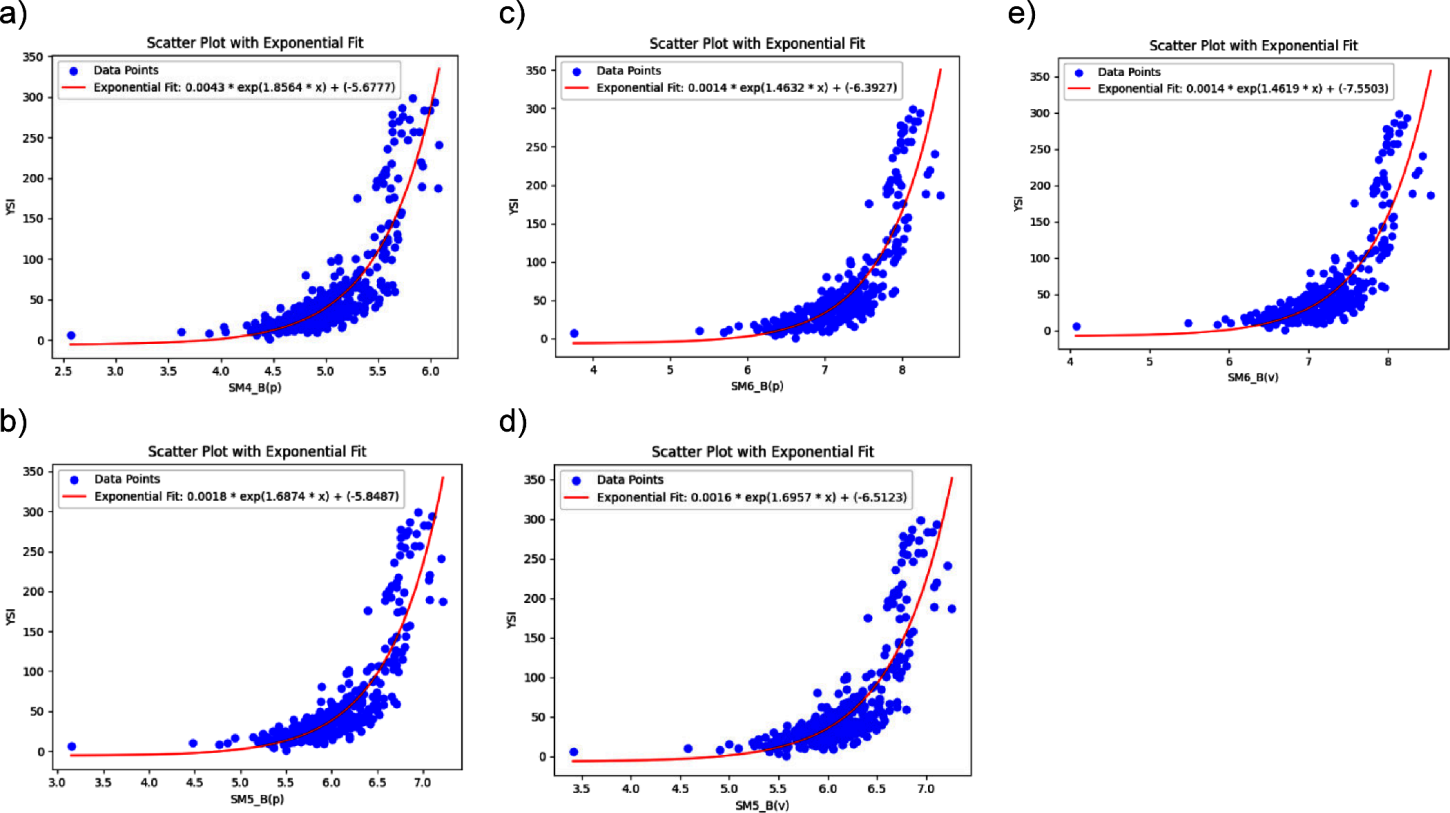
Exponential fit of YSI with (a) SM4_B­(p), (b) SM5_B­(p), (c) SM6_B­(p), (d) SM5_B­(v), and (e) SM6_B­(v) and YSI.

The scatter plots in Figures 8a to 8e provide empirical evidence supporting the use of exponential relationships. Figure 8a demonstrates a strong exponential trend between SM4_B­(p) and YSI, highlighting this descriptor’s high predictive value. Similarly, Figures 8b and 8c confirm the predictive relevance of SM5_B­(p) and SM6_B­(p), respectively. In contrast, Figures 8d and 8e show that while the van der Waals volume-weighted descriptors, SM5_B­(v) and SM6_B­(v), also follow exponential trends, they display slightly higher variance. This difference in fit quality likely arises from the more variable nature of van der Waals interactions across molecular structures. Despite this variance, these descriptors remain useful for predicting YSI, though with slightly reduced precision compared to mass-weighted descriptors like SM4_B­(p) and SM5_B­(p). This highlights the need to carefully select descriptors based on the specific molecular property being modeled, as different descriptor types can complement each other in representing physical properties.^42,43^

Incorporating both piPC and 2D matrix-based descriptors significantly enhances the robustness of the predictive model. Specifically, piPC descriptors provide detailed insights into the molecular pathways influencing YSI, while the 2D matrix-based descriptors capture more global structural features. This dual approach improves the model’s accuracy by addressing both local and global molecular characteristics. The integration of multiple descriptor types reduces the likelihood of overfitting, as the model is not reliant on a single type of molecular feature. Instead, it benefits from a more comprehensive representation of molecular structure, leading to better generalizability across different molecules. This is supported by Naseer et al.^44^ who highlighted the importance of using diverse descriptor sets to enhance model robustness and predictive performance. In brief, the combination of piPC descriptors and 2D matrix-based descriptors enhances the model’s ability to predict YSI accurately across a range of molecular structures. By leveraging the strengths of both local and global molecular representations, the model achieved greater precision and reliability, as evidenced by the exponential fits observed in the scatter plots. This approach not only augmented our understanding of the molecular interactions that lead to sooting tendencies but also provided a more robust foundation for future predictive modeling in this domain.

The integration of path count descriptors and 2D matrix-based descriptors, combined with advanced feature selection methods like the Genetic Algorithm, ensures a comprehensive representation of molecular structures that enhances the predictive power of the model. Interestingly, a major key finding of our research is the superior performance of the genetic algorithm (GA) model in reducing autocorrelation among features, which directly correlates with enhanced prediction accuracy. This outcome underscored the efficacy of sophisticated feature selection methods in refining model performance, which is a crucial aspect highlighted in recent literature. When juxtaposed with the works performed by Runzhao et al.,^45^ our approach underscored the potential limitations of deep learning models like convolutional neural networks, especially in computational efficiency. Despite their model’s superior feature extraction capabilities, our genetic algorithm-based model achieved a comparable balance of computational demand and predictive accuracy. This echoed the findings of Runzhao and co-workers, where ML-QSPR models were preferred over deep learning counterparts for their speed and accuracy.^46^

The multitask and transfer learning strategies explored in Larsson’s study^47^ resonated with our methodological advancements, particularly in enhancing prediction accuracy across a spectrum of fuel properties. Our approach aligns with this perspective, by demonstrating that advanced feature selection and ML techniques could substantially improve predictive models’ performance. However, unlike Larsson’s broad application across various fuel types, our approach concentrated on the intricacies of YSI prediction. Therefore, this offered a more focused yet profound understanding of sooting tendencies. Besides, Kessler et al.^39^ emphasized that the molecular structure’s role in sooting propensity, proposed practical equations for YSI prediction. To enable a direct comparison, we evaluated our pretrained QSPR-UOB 3.0 model (trained on all 558 compounds with the original 65/10/25 data split) on the 176 compounds (31.5%) overlapping with Kessler et al.’s data set without retraining. On this common subset, QSPR-UOB 3.0 achieved an RMSE of 7.02 YSI and an MAE of 6.14 YSI. Moreover, the use of artificial neural networks (ANNs) in Jameel^48^ to predict YSI for oxygenated fuels underlined the versatility and strength of ML techniques in fuel research. Our findings corroborated this perspective, and revealed that the genetic algorithm model not only exceled in feature reduction but also in maintaining high prediction accuracy across different fuel properties.

In comparison with previous sooting index prediction models, our approach demonstrates notable improvements in both accuracy and robustness. For instance, Kessler et al.^39^ reported a median absolute error of approximately 3 YSI units on an overlapping data set, whereas our QSPR-UOB 3.0 model achieved an R^2^ of 0.9993 with an RMSE of 7.567 YSI units. Moreover Jameel et al.^48^ obtained an R^2^ of 0.99 with an average prediction error of 3.4%. For uniformity, we computed QSPR-UOB 3.0s mean absolute percentage error on its test split, obtaining 8.9%. Our model’s performance indicates a further reduction in error and a higher degree of stability across multiple data splits. These improvements are primarily attributable to our dual feature selection strategy, which combines Gini importance with genetic algorithm optimization, and our rigorous uncertainty estimation framework. Together, these methodological advancements not only yield lower prediction errors but also provide reliable confidence measures, thereby enabling more informed screening and design of sustainable fuel formulations with reduced sooting tendencies.

In this study, the hybrid feature selection approach, which combined Genetic Algorithm and Gini Importance, has proven to be highly effective in reducing feature autocorrelation and enhancing model performance for predicting complex properties like the yield strength index. Compared to traditional methods such as random forest and principal component analysis, this hybrid approach presents clear advantages, particularly in handling high-dimensional, nonlinear relationships inherent in molecular systems. Furthermore, traditional methods like random forest have been widely recognized for their capacity to handle high-dimensional data while effectively selecting relevant features, even in the presence of correlations.^32^ However, a key limitation of random forest lies in its reliance on orthogonal splits, which can fail to capture complex nonlinear relationships.^49^ PCA, while powerful for dimensionality reduction, often overlooks critical nonlinear correlations, leading to potential losses in relevant information. Our hybrid approach, integrating GA with Gini Importance, effectively overcomes these limitations by optimizing feature selection through an evolutionary process. This allows the model to balance dimensionality reduction with the retention of features essential for capturing nonlinear relationships, enhancing the overall predictive performance. These results align with findings from Bader-El-Den and co-workers,^50^ who noted that GA’s capacity to iteratively refine feature sets enabled it to outperform more conventional methods in data sets involving high-dimensional interactions. This advantage is also evident in other domains, such as IoT network analysis^51^ and medical prediction tasks like Parkinson’s disease diagnosis,^52^ where hybrid methods have been shown to significantly enhance classification accuracy and precision. In our study, the GA-based model similarly outperformed the Gini model, particularly by reducing feature redundancy, which directly improves model efficiency and interpretability. A notable observation from our study is that feature counts for properties like CN, MON, KV, and IT were reduced by 30–45% using GA, without sacrificing predictive accuracy. This is critical in high-dimensional data sets such as those found in QSPR modeling, where reducing feature redundancy is essential for improving interpretability and computational efficiency. This finding echoes work in biomarker discovery, where hybrid feature selection methods have been shown to improve model reproducibility and biological plausibility by reducing redundant features.^53^ This reduction in features suggests that GA can optimize feature selection while still capturing the critical relationships between molecular descriptors and properties like YSI. However, it is essential to stress the trade-offs associated with the GA approach, especially regarding computational cost. The iterative nature of GAs is computationally intensive, requiring more resources than simpler methods like random forest or PCA. This trade-off is supported by findings from Richeldi and co-workers,^54^ who highlighted that while GA can optimize feature selection more effectively, its computational complexity can be a limiting factor in large-scale applications. Nevertheless, recent advancements in hybrid feature selection processes have made GA more efficient, making it increasingly applicable to large-scale predictive models, especially in fields like fuel property prediction. In addition, one of the unexpected findings in this study was the behavior of MON predictions, where despite having more samples than KV, MON exhibited a lower R^2^ value. This result diverges from typical expectations, where larger data sets usually provide more robust predictions. This anomaly suggests that factors beyond sample size, such as the inherent complexity of the target property, play a critical role in model performance, a point highlighted by Bader-El-Den et al.^50^ The nonlinear nature of certain fuel properties, like MON, required more advanced feature selection methods like GA to capture the intricate molecular interactions that simpler models might miss.^55^ Thus, future research should explore how these complexities affect model accuracy and whether further optimizations in feature selection can address such challenges.

The combined use of path count (piPC) and 2D matrix-based descriptors (SM4, SM5, SM6) for improving predictive accuracy in QSPR models. The combination of these descriptor types capitalizes on their complementary strengths, allowing the model to capture both local connectivity and global structural features of molecules, which is essential for predicting complex properties like YSI. The synergy between path count descriptors and 2D matrix-based descriptors has been well-documented.^56,57^ Furthermore, path count descriptors, such as piPC04 and piPC05, are computationally efficient and provide valuable insights into molecular connectivity, while matrix-based descriptors offer a more comprehensive view of molecular topology, mass, and electron distribution. Our findings, consistent with Gao et al.^58^ demonstrated that combining these descriptors improved the model’s ability to predict YSI more accurately than either type alone. This dual descriptor approach could be highly beneficial in future research on fuel properties, as it allows for a more holistic representation of molecular interactions. Moreover, the study’s use of exponential models to capture nonlinear relationships between descriptors and YSI proved to be a key factor in enhancing predictive accuracy. The preference for exponential fits, as demonstrated in this study, aligns with the literature, where nonlinear models are often superior for capturing the rapid changes in molecular properties.^59^ Studies like those by García-Jacas et al.^60^ further support the use of exponential models in molecular property prediction, highlighting their effectiveness in handling nonlinear behaviors that are prevalent in complex systems such as fuels.

The implications of this study extend beyond YSI predictions to other critical fuel properties such as viscosity, octane number, and sooting propensity. By demonstrating the effectiveness of GA-based models and the importance of combining multiple descriptor types, this study offers a roadmap for future research in fuel property prediction. As the field of fuel research evolves, particularly with the increasing reliance on machine learning and advanced computational techniques, the lessons from this study underscore the need for hybrid approaches that balance dimensionality reduction, feature selection, and predictive accuracy. Future research should focus on optimizing GA-based models to reduce computational costs while retaining their predictive power. This includes exploring how GA can be integrated with other machine learning methods, such as deep learning and support vector machines, to handle the growing complexity of fuel data sets. Additionally, the use of more sophisticated molecular descriptors, combined with exponential models, can further enhance the accuracy of predictions in fuel-related studies, providing valuable insights for the development of cleaner and more efficient fuels. In conclusion, this study demonstrates the significant advantages of using a hybrid GA-based approach for feature selection in QSPR models. By refining feature selection, employing diverse molecular descriptors, and leveraging exponential fits for nonlinear interactions, we provide a robust methodology for future predictive models in fuel research. These insights are not only applicable to YSI predictions but also offer valuable lessons for a wide range of fuel properties, paving the way for more accurate and computationally efficient fuel property predictions in future studies.

## 4 Conclusions

This study successfully developed and analyzed two predictive models for the YSI, demonstrating that machine learning approaches, particularly those incorporating advanced feature selection methods like genetic algorithms, can significantly enhance the accuracy of fuel property predictions. Our findings contribute to a deeper understanding of the molecular factors influencing soot formation, which is pivotal for the development of cleaner and more efficient fuels. Indeed, the models demonstrated substantial proficiency in predicting fuel properties such as yield sooting tendency, kinetic viscosity, ignition temperature, cetane, and octane numbers. Notably, the genetic algorithm model outshined the Gini importance model by efficiently reducing autocorrelated features, which enhanced its predictive accuracy. The detailed examination of feature importance has yielded a deeper understanding of the molecular descriptors that significantly impact YSI, and highlighted the importance of 2D matrix-based descriptors. A generalized equation for YSI prediction, derived from these descriptors, marked a significant advance in computational fuel property prediction, which paved the way for future research and development in fuel technology.

Model training insights, that were obtained from learning curves, provided an understanding of the models’ convergence and generalization capabilities, and illustrated a decent balance between underfitting and overfitting. The analysis demonstrated the importance of data preprocessing in refining model performance, which is crucial for handling complex problems effectively. Hyperparameter optimization emerged as a key factor in enhancing model architecture and performance, whereas the use of conformal prediction for uncertainty estimation lent additional credibility to the predictions by providing a measure of confidence. The study also revealed the essential task of balancing predictive efficiency against error rates. Indeed, this remains a challenge that shows the nuanced reality of practical model application.

Moreover, our investigation emphasized the importance of judicious model selection, which weighed the trade-offs between complexity and accuracy, and recognized the inherent limitations of each modeling approach. This work illuminated the critical role of predictive modeling in fuel property analysis, and advocated for rigorous preprocessing, hyperparameter optimization, and uncertainty estimation to forge robust and reliable predictions. Ultimately, this approach contributed essential insights into the field of fuel property analysis, and underlined the necessity of a sophisticated method to modeling that takes into account accuracy, efficiency, and error dynamics. As the quest for sustainable and efficient fuel alternatives advances, our findings offer valuable guidance for future endeavors in energy research, influence strategic decisions and foster innovations in the development of cleaner, and more efficient fuel solutions.

## Supplementary Material





## Data Availability

The data required to reproduce these findings can be found in the Supporting Information.

## Notes

Physicochemical properties that affect engine performance (PDF)
Dataset of YSI (XLSX)
