# Supplementary material for: Predictive Modeling of Yield Sooting Index Using Machine Learning with Uncertainty Estimation
Source: ACS Omega. 2025 Jun 10;10(24):25336–49. doi: 10.1021/acsomega.5c00042 (PMC12199018; doi:10.1021/acsomega.5c00042)
Supplement: Supplementary file 1 [file ao5c00042_si_001.pdf]

Predictive modeling of Yield Sooting Index using machine learning with uncertainty estimation

Zied Hosni[a*], Xike Chen[a], Sofiene Achour[b,c], Fatma Saadi[d]

[a] Institute for Materials Discovery, University College London, Gower Street, London, WC1E 6BT, United Kingdom

[b] University of Tunis El Manar, Research Unit of Modeling in Fundamental Sciences and Didactics, IPEIEM, PO Box 254, El Manar 2, 2096, Tunis, Tunisia

[c] Center for Research in Microelectronics and Nanotechnology (CRMN), Technopôle de Sousse "Novation City", Sahloul (BP 334 Sahloul Sousse 4054), Tunisia

[d] Department of Chemistry, College of Science, Northern Border University, Arar, Saudi Arabia.

* Email: z.hosni@ucl.ac.uk

Table S1. Physicochemical properties that effect engine performance.

Category	Parameter	Symbol
volatility specification	melting point	T_m
	boiling point	T_b
	vapor pressure	VP
	enthalpy of vaporization	ΔH_{vap}
atomization specification	surface tension	γ
	kinematic viscosity	ν
energy density	lower heating value	LHV
sooting tendency	liquid density	ρ
	yield sooting index	YSI
ignitability	cetane numbers	CN
	research octane numbers	RON
	motor octane numbers	MON
	ignition temperature	IT
	flash point	FP
	lower/upper flammability limits	LFL/UFL
